# The impact of hand hygiene in the primary care: to go beyond the hospital setting

**DOI:** 10.1186/2047-2994-4-S1-P161

**Published:** 2015-06-16

**Authors:** I Devesa, I Neves, D Peres, F Vieira, G Poças

**Affiliations:** 1 Infection Control and Antimicrobial Resistance Unit, Unidade Local de Saúde de Matosinhos, Matosinhos, Portugal; 2 Infectious Diseases Unit, Unidade Local de Saúde de Matosinhos, Matosinhos, Portugal; 3 Primary Care Unit, Unidade Local de Saúde de Matosinhos, Matosinhos, Portugal

## Introduction

Scientific evidence shows that hand hygiene is an easy and efficient practice in the reduction of healthcare associated infection, contributing to a decrease in morbidity and mortality. World Health Organization (WHO) “SAVE LIVES: Clean Your Hands” (SL-CYH) is still very centered in the hospital/ long term care settings.

## Objectives

Adaptation of the WHO SL-CYH campaign to the primary care setting.

## Methods

Implementation of the WHO multimodal methodology in a primary care setting involving 13 units, serving a population about 110.000 inhabitants. The implementation of the campaign was coordinated by the Infection Control and Antimicrobial Resistance Unit and focused on the following actions: (1) audits and correction measures to structural conditions of the units (for example: existence of alcohol-based handrub – ABHR- dispensers and washbasins to hand hygiene) and pocket ABHR available to the home care; (2) infection control link professionals observer training (replicated in each unit) and subsequent observational study before/after the campaign in 2013 and the year after (2014); (3) campaign launch event in each unit with distribution of promotional material (pens, pins, flyers, posters, small films, didactic games) and signature of formal commitment by the professionals; (4) celebration of Hand Hygiene Day (5 of May) with a walk for the general population and involving the local and national media; (5) organization of hand hygiene poster competition for professionals and (6) participation in the E-bug project directed to schools.

## Results

Compliance rates on hand hygiene in the participating units was 56%, 82% and 82% before, after campaign in 2013 and 2014, respectively. The rate of compliance in the “first moment” was of 46%, 61% and 71% before, after campaign in 2013 and 2014, respectively.

## Conclusion

The campaign methodology of WHO SL-CYH showed to be successful when applied to the primary care setting. The biggest difficulty was the inexistence of specific material in portuguese language and directed to this setting. The community intervention level seems to be an interesting contribution to WHO SL-CYH campaign.

## Disclosure of interest

None declared.

